# Monitoring mosquito nuisance for the development of a citizen science approach for malaria vector surveillance in Rwanda

**DOI:** 10.1186/s12936-020-03579-w

**Published:** 2021-01-10

**Authors:** Marilyn Milumbu Murindahabi, Willem Takken, Xavier Misago, Elias Niyituma, Jackie Umupfasoni, Emmanuel Hakizimana, Arnold J. H. van Vliet, P. Marijn Poortvliet, Leon Mutesa, Nathalie Kayiramirwa Murindahabi, Constantianus J. M. Koenraadt

**Affiliations:** 1 Laboratory of Entomology, Wageningen University & Research, Wageningen, The Netherlands; 2 College of Sciences and Technology, University of Rwanda, Kigali, Rwanda; 3 Malaria and Other Parasitic Diseases Division, Rwanda Biomedical Center, Kigali, Rwanda; 4 Environmental Systems Analysis Group, Wageningen University & Research, Wageningen, The Netherlands; 5 Strategic Communication Group, Wageningen University & Research, Wageningen, the Netherlands; 6 College of Medicine and Health Sciences, University of Rwanda, Kigali, Rwanda

**Keywords:** Malaria, Perceived mosquito nuisance, House features, Livestock, Surveillance, Culicidae, *Anopheles*

## Abstract

**Background:**

Many countries, including Rwanda, have mosquito monitoring programmes in place to support decision making in the fight against malaria. However, these programmes can be costly, and require technical (entomological) expertise. Involving citizens in data collection can greatly support such activities, but this has not yet been thoroughly investigated in a rural African context.

**Methods:**

Prior to the implementation of such a citizen-science approach, a household entomological survey was conducted in October–November 2017 and repeated one year later in Busoro and Ruhuha sectors, in southern and eastern province of Rwanda, respectively. The goal was to evaluate the perception of mosquito nuisance reported by citizens as a potential indicator for malaria vector hotspots. Firstly, mosquito abundance and species composition were determined using Centers for Disease Control and Prevention (CDC) light traps inside the houses. Secondly, household members were interviewed about malaria risk factors and their perceived level of mosquito nuisance.

**Results:**

Tiled roofs, walls made of mud and wood, as well as the number of occupants in the house were predictors for the number of mosquitoes (Culicidae) in the houses, while the presence of eaves plus walls made of mud and wood were predictors for malaria vector abundance. Perception of mosquito nuisance reported indoors tended to be significantly correlated with the number of *Anopheles gambiae *sensu lato (*s.l*.) and Culicidae collected indoors, but this varied across years and sectors. At the village level, nuisance also significantly correlated with *An. gambiae s.l.* and total mosquito density, but only in 2018 while not in 2017.

**Conclusions:**

Perception of mosquito nuisance denoted in a questionnaire survey could be used as a global indicator of malaria vector hotspots. Hence, involving citizens in such activities can complement malaria vector surveillance and control.

## Background

Malaria remains a public health concern in Rwanda despite the gains made in malaria reduction in the past decades [1]. Since 2012, malaria has increased every year, thereby impeding the progress made up to 2011 [2]. From 2012 to 2016, the country reported an eight-fold increase in malaria cases. Additionally, malaria-related deaths increased from 325 in 2012 to 663 in 2016. The eastern and southern parts of the country have been the most afflicted regions. The increase in malaria cases has been observed in all districts, including districts that were previously defined as being at pre-elimination phase. This increase was observed across all age groups, suggesting the entire population is at risk of acquiring a malaria infection [2].

In Rwanda, malaria vector surveillance is carried out monthly in 12 sentinel sites across the country. It aims to monitor key parameters of malaria vectors, and provides entomological data, such as entomological inoculation rate (EIR), to guide the planning of vector control interventions [3, 4]. At present, the monitoring of malaria mosquito density is combined with malaria incidence and helps to determine the spatio-temporal spread of infections. However, active mosquito surveillance is conducted in only 3% (12/416) of the sectors of Rwandan territory, while in the remaining 97%, mosquito monitoring is not implemented [5]. The current mosquito surveillance approach is challenging to be implemented in all areas of the country due to the inadequate local capacity in entomology and the high costs related to extending vector surveillance countrywide, hence alternative approaches are desired [3].

Passive surveillance through citizen-science is a tool to track mosquito presence and spread, such as of *Aedes albopictus*, a potential vector of dengue, Zika and chikungunya viruses [6, 7]. Studies carried out in The Netherlands and Spain showed that citizens provided scientifically valuable information through questionnaires and through sending mosquito samples to laboratories in charge of mosquito identification. This can ease cost constraints for mosquito surveillance [6, 8]. Mosquito nuisance reported by citizens via a questionnaire in combination with actual mosquito samples collected by citizens revealed the presence or absence of two known biotypes of *Culex pipiens* and their hybrids, which can be important vectors of West Nile virus [8].

In the current study, prior to the implementation of a wider citizen-science programme for malaria vector surveillance in Ruhuha, Rwanda, mosquito species composition was determined using a conventional mosquito trapping method in two rural sectors by means of two cross-sectional surveys performed in 2017 and 2018. In addition, factors that could explain the observed spatial distribution of mosquito species collected were analysed. Lastly, it was investigated whether perceived mosquito nuisance reported by the participants could provide an indication of potential (malaria) vector hotspots, especially in areas where entomological surveillance for malaria vectors is not implemented.

## Methods

### Study site

**Fig. 1:**
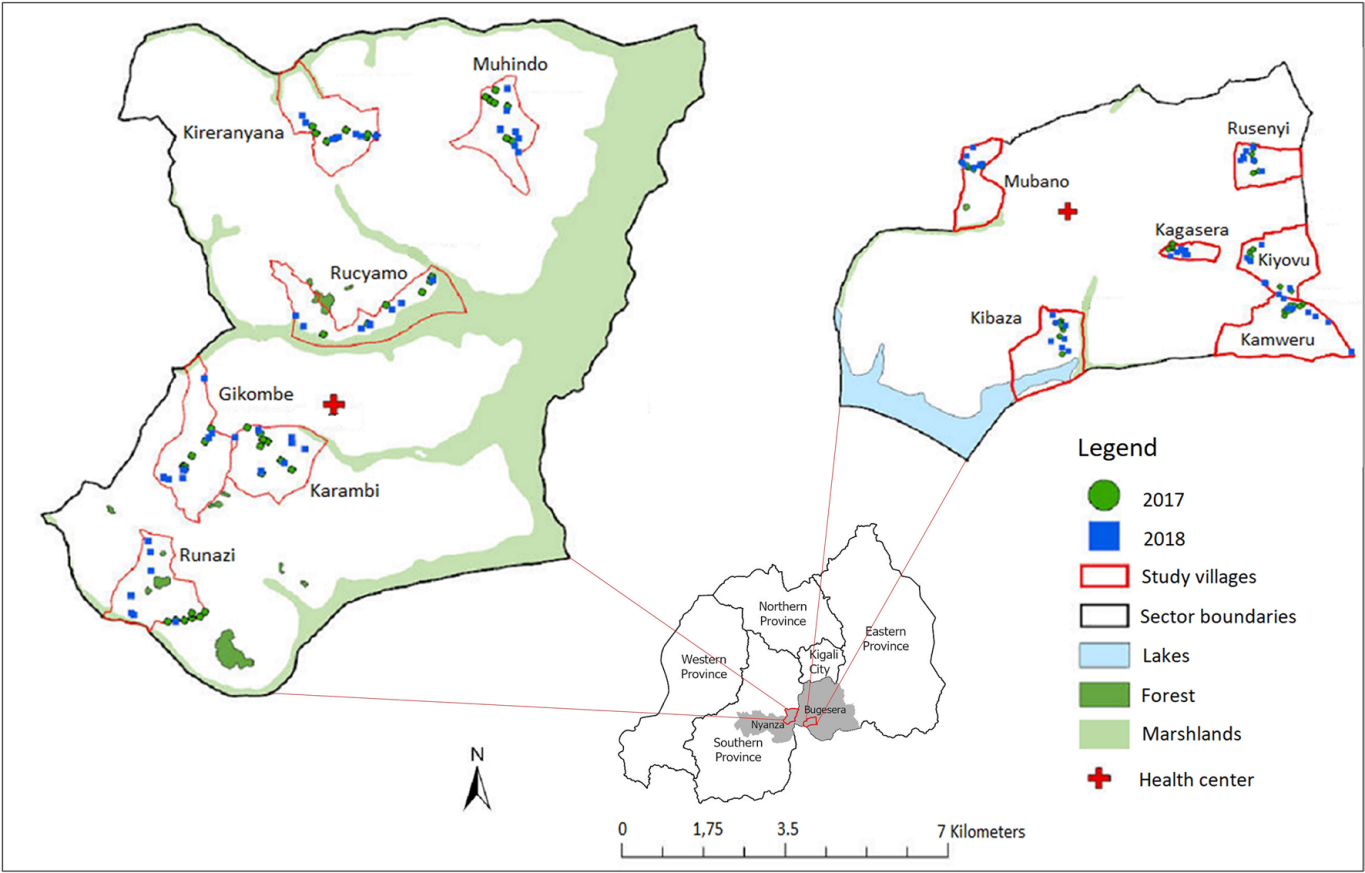
Maps of Busoro and Ruhuha sectors located in Bugesera and Nyanza districts (in grey) showing houses randomly selected (blue and green dots for the different years of study) from six villages that were selected for the household survey and mosquito survey using Centers for Disease Control and Prevention miniature light traps (CDC-LT)

Household and entomological surveys were carried out in Ruhuha and Busoro sectors, located respectively in Bugesera district (eastern province), and in Nyanza district (southern province) in Rwanda (Fig. 1). The choice of the study sites was based on the large number of malaria cases reported in Ruhuha since 2012, and the long-term working relationship with the local health centre. Ruhuha sector covers 54 km^2^ and is sub-divided into 35 villages [9]. An estimated 24,000 people are living in more than 5100 households (HHs) [10]. The area is a high malaria-endemic zone [11]. Busoro is a sector covering 74 km^2^ and is sub-divided into 41 villages. It has a total population of approximately 34,000 people living in 8,000 HHs [12]. Irrigated rice fields are the main type of land use in both sectors. Both sectors differ from each other by the area of irrigated rice fields which is 40% larger in Busoro (178 hectares or 2.4% of the land surface) in comparison with Ruhuha (93 hectares or 1.7% of the land surface) [13, 14]. Additionally, Ruhuha is located near the shores of Lake Cyohoha South [15]. The wetlands potentially serve as favourable places for mosquito breeding.

### Study design

Household and entomological surveys were conducted in Ruhuha and Busoro for three weeks in October–November 2017 and were repeated in a modified form in the same period in 2018. Data from the household survey were coupled with entomological data collected for both sectors and years. In addition to the demographic characteristics, factors were defined that could explain the variation in mosquito abundance. Additionally, the relationship between perceived mosquito nuisance experienced by the citizens in their house and mosquitoes collected using CDC light traps in Busoro and Ruhuha was assessed.

### Household selection

The studied HHs were selected among those that were part of a larger household survey [12]. Six villages per sector were selected by simple random sampling. In Busoro sector, the villages of Kireranyana, Gikombe, Muhindo, Karambi, Runazi and Rucyamo were selected while in Ruhuha sector, Kagasera, Kibaza, Kiyovu, Kamweru, Rusenyi, and Mubano villages were selected for the study (Fig. 1). At the village level, lists of HHs were provided by the village leaders, and a systematic random sampling was used to draw a sample of HHs to be visited. As a result, 30 to 31 HHs were selected for each village for the large household survey. From these HHs, 6–8 HHs were selected for the household survey. In 2017, 82 HHs were thus selected randomly (42 from Busoro and 40 from Ruhuha) and considered for the study. In 2018, the same survey was repeated by selecting randomly and directly from the list, excluding HHs during the previous survey in 2017. In other words, HHs interviewed in 2017 were different from those from 2018. In 2018, 84 HHs were selected, 42 from Busoro and 42 from Ruhuha. If household members were absent at the time of the interview, the interviewers progressed to the next selected house. Only residents above 18 years of age and who consented to participate in the study were interviewed. The house occupied by the interviewed resident was selected for the mosquito collection after obtaining informed consent.

### Data collection

#### Household survey

For the household survey conducted in 2017 and 2018, perception of mosquito nuisance experienced indoors and per season was assessed by the citizens in Busoro and Ruhuha. Six to 8 household surveys were carried out per day. The average interview time was 45 min up to one hour per household. The questions were originally written in English and translated into the local language (*Kinyarwanda*), and prior to the survey translated back into English. The questionnaire was pre-tested in a pilot study of 10 HHs selected randomly in the neighbouring Mareba sector, eastern province, for its consistency, and then revised consequently. Field data collectors were trained in addressing the questionnaire before conducting the survey. Only participants who consented were interviewed in *Kinyarwanda*. All translations were made by professional translators including members of the project team and cross-checked by native speakers. The translators were asked to review and cross-check the items and identify any problems in wording, terminology, ability to understand, and relevance.

Questionnaire data were collected in electronic forms using Open Data Kit (ODK) Collect set-up [12, 16]. The questionnaire encompassed questions with closed- and open-ended questions. The questionnaire contained different sections with questions related to demographic data of the participants, perception of mosquito nuisance and its seasonality, knowledge on malaria, and to vector and malaria control practices. For statistical analysis, data on demographic characteristics, household characteristics and mosquito nuisance perceived indoors by the respondents were used.

#### Demographic characteristics

Demographic data included gender, age, marital status, education level, occupation, *Ubudehe* category (a community-based social categorization of household and dependents into different groups based on their income) [17], size of the household, bed net ownership, and mobile phone ownership of the participants. Other characteristics, such as house features (type of wall (mud/clay or wood), type of floor (cement or mud), type of roof (iron or tile sheets), presence of eaves, and livestock ownership (species owned and location where they were kept) were also included in the questionnaire.

#### Perception of mosquito nuisance

Respondents were asked to answer the question whether they experienced mosquito nuisance in their environment. Participants who experienced mosquito nuisance were requested to scale the level of nuisance experienced in the house on a 6-point Likert scale (0 = no nuisance, 1 = very little to 5 = very much). They were also requested to indicate when (rainy or dry seasons) they experienced mosquito nuisance.

#### Mosquito collection by CDC light traps

Mosquitoes were collected in 2017 and 2018 in 166 selected houses in Ruhuha (82) and Busoro (84) among the interviewed participants using a miniature CDC light trap. The CDC light traps were set up in the bedroom and hung at the foot end of the bed, with the shield of the trap at 1.5 m from the floor [18]. The traps were set up at 18:00, and the owner of the room was instructed to put off the light of the trap and tie the bag connected to the collecting cup at 06:00 the next morning to avoid mosquitoes escaping from the traps. After their collection from the traps, mosquitoes were stored in labelled petri dishes before morphological identification in the laboratory.

#### Mosquito identification

Mosquitoes were identified using standard morphological identification keys for anophelines and culicines [19, 20]. Mosquitoes were scored as unfed or blood fed. The mosquitoes were then stored under silica gel in labelled Eppendorf tubes with the codes of the respondent interviewed and kept in an envelope with the name of the village under cool conditions at the central Laboratory of Entomology for identification of *Plasmodium falciparum* infection and further molecular species identification.

### Laboratory processing

#### Blood meal identification

For samples collected in 2017, each mosquito abdomen was ground in 100 µl of phosphate-buffered saline (PBS), and then filled up to 1 ml PBS. Blood meals were identified by direct enzyme-linked immunosorbent assay (ELISA) using antihost (IgG) conjugate against human and cow proteins in a single-step assay [21]. The non-reacting samples were then tested subsequently using goat IgG. ELISA results were read visually [22]. The anthropophilic rate was determined as the proportion of mosquitoes that fed exclusively on human blood among all fed mosquitoes.

#### Sporozoite rates

The head and thorax of all female *Anopheles gambiae *sensu lato (*s.l*.) collected in 2017 using CDC light traps were used to test for the presence of circumsporozoite protein (CSP) of *P. falciparum* using ELISA. A sample with an optical density (OD) value above the cut-off (cut-off = 2 × mean OD of 7 negative samples) was considered positive [23]. The sporozoite rate was calculated as the number of mosquitoes infected with *P. falciparum* sporozoites divided by the total number of mosquitoes processed.

#### Molecular species identification

For molecular identification of the sibling species of *An. gambiae s.l.*, a random sample of 9% (n = 233/2514) of the total number of *An. gambiae s.l.* collected during the two years and from both sectors were identified using the rDNA-polymerase chain reaction (PCR) assay [24]. If the initial PCR testing failed to amplify a sample, then the PCR analysis was repeated once or twice until successful amplification was achieved. If a sample could not be identified after three rounds of PCR, it was scored as unknown [25].

### Data analysis

Household survey data were imported from ODK software into Microsoft Excel (2016) and checked for consistency in the values and answers. They were electronically loaded onto a central server for backup, translated and coupled with data from the entomological survey. Statistical analysis was undertaken in SPSS 23.0 (SPSS Inc, Chicago, IL, USA), and included the calculation of frequencies and Chi-squares statistics. Bivariate analysis of correlation between the dependent variable (number of *An. gambiae* s.l. or Culicidae) and the independent variables (number of members of the household, house structural features (floor, wall, roof), species of animal kept in the house and presence of eaves was performed to determine predictors that could explain the mosquito abundance indoors. Only predictors that had a screening significance lower than 0.1 were then considered for the final models. For this purpose, generalized linear models (GLM, negative binomial with log link) were used. Besides house structural features, household size and livestock ownership, other factors included in the GLM were sector (Ruhuha/Busoro) and the year of study (2017/2018). All data on mosquito collections were entered into Excel to calculate the sporozoite rate and human blood index and the various mosquito species identified were summarized as proportions. Furthermore, Spearman correlation analysis was used to analyse the relationship between mosquito nuisance reports by the respondents and number of mosquitoes and species collected in the respondents’ houses.

## Results

### Demographic characteristics

One-hundred and sixty-six respondents were enrolled both in the household survey and mosquito collection, out of 167 (n = 166/167, 99%) requested respondents to participate. Table 1 provides an overview of the demographic characteristics of the participants. Overall, both sectors were similar in their demographics. In both sectors more than half of the respondents were female (64%). The average age of the respondents in Busoro was 44 years (n = 84), and 41 years (n = 82) in Ruhuha. Almost 76% of the respondents were schooled (no category) while the remainder (24%) was unschooled. Respondents from Busoro were more highly educated (81%) than those from Ruhuha (71%). Most of the respondents were farmers (93%), followed by self-employed (1%), private officer (1%), student (1%), and unemployed (4%). However, more respondents in Busoro than in Ruhuha were farmers (98 *vs* 89%). In the latter sector, more individuals were unemployed. There was no significant difference when comparing *Ubudehe* categories (a community-based social categorization of household and dependents into different groups based on their incomes) between the two sectors. Most of the participants owned a bed net (74%), but there was a significant difference in bed net ownership between Busoro and Ruhuha (81 vs 66%). Almost half of the participants owned a mobile phone (46%). There was no significant difference in mobile phone ownership when comparing both sectors.

**Table 1:** Demographic and household characteristics of 166 respondents in Busoro and Ruhuha sector, Rwanda

Variables	Busoro, n (%)	Ruhuha, n (%)	Total	*P*
Gender				
Male	38 (45)	22 (26)	60 (36)	
Female	46 (55)	60 (73)	106 (64)	0.014
Age				
19–24	7 (8)	8 (10)	15 (9)	
25–44	31 (37)	42 (51)	73 (44)	
45–59	35 (42)	23 (28)	58 (35)	
> 60	11 (13)	9 (10)	12 (12)	0.222
Marital status				
Never married	4 (5)	7 (9)	11 (7)	
Married	40 (48)	36 (44)	76 (46)	
Living together	16 (19)	19 (23)	35 (21)	
Separated (Divorce)	11 (13)	5 (6)	16 (10)	
Widow	13 (16)	15 (18)	28 (17)	0.444
Education				
None	16 (19)	24 (29)	40 (24)	
Incomplete primary	43 (51)	31 (38)	74 (45)	
Primary	15 (18)	19 (23)	34 (21)	
Incomplete secondary	5 (6)	3 (3)	8 (5)	
Secondary	5 (6)	5 (5)	10 (5)	0.342
Occupation				
Farmer	82 (98)	73 (89)	155 (93)	
Self-employed	0 (0)	2 (2)	2 (1)	
Private officer	1 (1)	0 (0)	1 (1)	
Student	0 (0)	1 (1)	1 (1)	
Unemployed	1 (1)	6 (4)	7 (4)	0.088
*Ubudehe* category				
Category 1	12 (14)	16 (20)	28 (17)	
Category 2	37 (44)	34 (42)	71 (43)	0.666
Category 3	35 (42)	32 (39)	67 (36)	
Size of the household				
1 to 2	9 (11)	9 (11)	18 (11)	
3 to 5	47 (56)	48 (58)	95 (57)	
≥ 6	28 (33)	25 (31)	53 (32)	0.925
Bed net ownership				
No	16 (19)	28 (34)	44 (27)	0.028
Yes	68 (81)	54 (66)	122 (74)	
Mobile phone ownership				
No	45 (54)	45 (55)	90 (54)	0.866
Yes	39 (46)	37 (45)	76 (46)	

*P-*values are based on Chi-square analysis of the proportions between the two sectors

### House features

Most of the participants had mud floor houses (83%) with mud walls (59%), closed eaves (65%), and iron sheet roofing (69%) (Table 2). There were no significant differences between both sectors for house features (eaves presence, floor, wall features), except for iron sheeting (100 in Ruhuha *vs* 38% in Busoro) (Table 2).

**Table 2:** House features of the 166 respondents in Busoro and Ruhuha sector, Rwanda

Variables	Busoro, n (%)	Ruhuha, n (%)	Total, n (%)	*P*
House features				
Eaves				
No	55 (65)	53 (64)	108 (65)	
Yes	29 (35)	29 (36)	58 (35)	0.909
Floor				
Cement	13 (15)	16 (19)	29 (18)	0.494
Mud/clay	71 (85)	66 (81)	137 (83)	
Roof				
Iron sheets	32 (38)	82 (100)	114 (69)	
Tile sheets	52 (62)	0 (0)	52 (31)	< 0.0001
Wall				
Mud/clay	47 (56)	51 (62)	98 (59)	
Wood and mud	37 (44)	31 (38)	68 (41)	0.414

*P-*values are based on Chi-square statistical comparisons

### Livestock ownership

Overall, there was a significant difference in livestock ownership when comparing both sectors (*P* = 0.029). Of the respondents, 72% owned at least one species of livestock (cows, pigs, poultry, rabbits, goats or sheep; Table 3). However, participants in Busoro owned more livestock than participants in Ruhuha (80 vs 65%). Goats were the most often owned livestock. Poultry ownership differed significantly between Busoro and Ruhuha (*P* = 0.003): respondents in Busoro owned more poultry indoor than Ruhuha.

**Table 3:** Proportion of households keeping each species of livestock indoors and/or outdoors in Busoro and Ruhuha sector, Rwanda

	Busoro			Ruhuha			*P*
	Indoor, n (%)	Outdoor, n (%)	Household without livestock	Indoor, n (%)	Outdoor, n (%)	Household without livestock	
Cow	0 (0)	33 (49)	34 (51)	0 (0)	28 (53)	25 (47)	0.697
Pig	0 (0)	6 (9)	61 (91)	0 (0)	5 (9)	48 (91)	0.928
Poultry	25 (37)	5 (8)	37 (55)	9 (17)	0 (0)	44 (83)	0.003
Rabbit	2 (3)	1 (2)	64 (96)	2 (4)	0 (0)	51 (96)	0.654
Goat	44 (66)	10 (15)	13 (19)	30 (57)	11(21)	12 (23)	0.572
Sheep	0 (0)	0 (0)	67 (100)	1(2)	0 (0)	52 (99)	0.259

*P-*values are based on Chi-square statistical comparisons

### Mosquito species composition by CDC light traps

From the mosquitoes collected in 2017 and 2018, 74% (n = 7370) were collected in 2017 (Additional file 1) and 26% (n = 2595) in 2018 (Additional file 2). Of all mosquitoes, 74.2% were morphologically identified as culicines and 25.2% as anophelines. Among female mosquitoes collected, 25.6% (n = 2210) were fed and 77.8% (n = 7755) were unfed. Of the total anophelines collected (n = 2514), 94.2% was *An. gambiae s.l*. Other *Anopheles* species collected included *Anopheles brohieri* (0.2%), *Anopheles funestus* (1.2%), *Anopheles maculipalpis* (1.1%), *Anopheles pharoensis* (0.6%), *Anopheles rufipes* (0.5%), and *Anopheles ziemanni* (2.2%). Busoro recorded 72.8% of the total *An. gambiae s.l.* collected, the remaining 27.2% was collected in Ruhuha. *Anopheles funestus* (n = 31), *An. pharoensis* (n = 14) and *An. ziemanni* (n = 56) were the most frequently encountered other human-biting *Anophele*s species identified in Busoro, while *An. brohieri* (n = 2),) *An. rufipes* (n = 13), and *An. maculipalpis* (n = 5) were *Anopheles* species known as non-human-biting. In Ruhuha, *An. pharoensis* (n = 1) was another human biting malaria vector, and *An. brohieri* (n = 2), *An. maculipalpis* (n = 22) were the non-human-biting anophelines. The malaria vector *An. funestus* was not collected in Ruhuha.

From 7451 culicines, 99.7% (n = 7425) consisted of *Culex* spp. and 0.3% (n = 26) of *Mansonia* spp. The highest proportion of culicines identified was *Cx. quinquefasciatus* (99.6%), with 89.2% (n = 6647) from Busoro and 10.4% (n = 778) from Ruhuha, respectively.

### Blood-feeding behaviour

Of 1,046 *Anopheles* spp. collected, 100 were selected randomly. Ninety-eight specimens were *An. gambiae s.l.*, while the other two were *An. maculipalpis* and *An. rufipes*, respectively. Of all 100 blood-fed specimens, 82 (82%) had fed on a single host (human, goat or bovine), while 5 (5%) had fed on mixed hosts and 13 were unspecified for the antigens assayed, suggesting that these *An. gambiae s.l.* had fed on other hosts than humans, goats or cattle (Table 4). For the remaining 87 *Anopheles*, 58% (n = 58) of the *An. gambiae s.l.* were engorged with human blood and 1% (n = 1) *An. gambiae s.l.* was engorged with blood of both human and goat origin.

**Table 4:** Host blood antigen detected in three mosquito species (October–November 2017)

Host blood	*An. gambiae s.l*	*An. maculipalpis*	*An. rufipes*	Total
	n (%)	n (%)	n (%)	
Human	58 (58)	0 (0)	0 (0)	58
Goat	2 (2)	1 (1)	0 (0)	3
Bovine	20 (20)	0 (0)	1 (1)	21
Goat and bovine	4 (4)	0 (0)	0 (0)	4
Human and goat	1 (1)	0 (0)	0 (0)	1
Unspecified	13 (13)	0 (0)	0 (0)	13
Total	98 (98)	1 (1)	1 (1)	100

### Plasmodium falciparum infection rates

Of the 1,046 mosquitoes tested by ELISA, *P. falciparum* CSP antigen was detected in 14 out of 1013 (1.3%), 11 out of 971 tested (1.1%) *An. gambiae s.l.* and 3 out of 42 tested *An. ziemanni* (7.1%). The overall sporozoite rate of anopheline species was 1.3%. The infection rate was 0.5% (3/573) in Busoro and 2.3% (11/473) in Ruhuha.

### Molecular identification of members of the Anopheles gambiae complex

Of the 9.4% (236/2514) of the *An. gambiae s.l.* selected for sibling species identification from 2017 and 2018, 145 (61.4%) were identified as *An. gambiae *sensu stricto (*s.s*.) and 74 (31.4%) as *Anopheles arabiensis* (Additional file 3). Sixteen (6.8%) were not amplified and one sample was contaminated (0.4%).

### Factors explaining mosquito abundance

After bivariate analysis, different species kept in the houses, such as poultry, rabbit, goat, and sheep (Table 3), did not show a statistically significant correlation (*P* < 0.1) with the dependent variables. Therefore, they were not selected for GLM analysis. The house features that included the materials used for the construction of the roof and the wall, and the numbers of occupants in the house, were predictors for the number of mosquitoes (Culicidae) in the houses. The predictors roof, wall and size of the household had a statistically significant effect on the number of mosquitoes indoors, while the floor composition did not contribute statistically to a difference (Table 5).

**Table 5:** Determinants of mosquito and malaria vector abundance

Variables	β	Incidence rate ratio	95% CI	*P*
Culicidae				
Sector				
Busoro	1.079	2.941	1.807–4.787	< 0.001
Ruhuha	*			
Year of study				
2017	0.928	2.528	1.742–3.668	< 0.001
2018	*			
Floor				
Cemented	− 0.309	0.734	0.421–1.280	0.276
Earthed	*			
Roof				
Iron sheets	− 0.846	0.429	0.253–0.729	0.002
Tile sheets	*			
Wall				
Mud/clay	− 0.913	0.401	0.273–0.591	< 0.001
Wood-Mud	*			
Family size				
Family size	0.153	1.166	1.058–1.284	0.002
*An. gambiae s.l.*				
Sector				
Busoro	1.223	3.399	1.913–6.038	< 0.001
Ruhuha	*			
Year of study				
2017	0.110	1.117	0.706–1.766	0.637
2018	*			
Eaves				
No eaves	− 0.560	0.571	0.361–0.904	0.017
Eaves	*			
Floor				
Cemented	− 0.393	0.675	0.359–1.271	0.223
Earthed	*			
Roof				
Iron sheets	0.425	1.529	0.844–2.771	0.162
Tiles sheets	*			
Wall				
Mud/clay	− 0.838	0.433	0.278–0.673	< 0.001
Mud and wood	*			

^*^Reference category

There were significantly more Culicidae in Busoro than in Ruhuha, and the incidence rate ratio for 2017 was 2.5 times that of 2018 (*P* < 0.001; Table 5). Houses with tiled roofs were more exposed to mosquitoes than houses with an iron roof (*P* = 0.002; Table 5). Likewise, houses with walls made of mud and wood had a larger number of mosquitoes (Culicidae) than houses with walls made with mud (*P* < 0.001; Table 5).

Similarly, for the total number of only *An. gambiae s.l*., there were more females collected in Busoro than in Ruhuha. However, the year effect was not significant (Table 5). Houses with closed eaves and mud walls were more likely to not have malaria vectors resting inside the house than houses with open eaves or walls made with wood and mud.

### Perceived mosquito nuisance per sector

In total, 96% (n = 159, both 2017 and 2018) of the respondents reported to have experienced at least some mosquito nuisance. There was a significant difference between both sectors in the proportion of respondents that reported mosquito nuisance (*P* = 0.050) (Table 6). Of those that did perceive nuisance, 6% (n = 10) experienced “very little”, 25% (n = 43) experienced “little”, 17% experienced “some”, 32% experienced “much” (n = 52, 32%) and 16% reported “very much” nuisance. There was no significant difference between the two sectors when comparing the level of mosquito nuisance experienced indoors (*P* = 0.177; Table 7).

**Table 6:** Perceived mosquito nuisance reported by 166 respondents in Busoro and Ruhuha

Variables	Busoro, n (%)	Ruhuha, n (%)	Total, n (%)	*P*
Nuisance				
No	1 (1)	6 (7)	7 (4)	
Yes	83 (99)	76 (92)	159 (96)	0.05
Total	84	82		

*P* is based on a Chi-square statistical comparison between sectors

**Table 7:** Perceived mosquito nuisance indoors for 2017 and 2018 in the study areas

Nuisance scale	Busoro, n (%)	Ruhuha, n (%)	Total, n (%)	*P*
No nuisance	1 (1)	6 (7)	7 (4)	
Very little	3 (4)	7 (9)	10 (6)	
Little	23 (27)	20 (24)	43 (25)	
Somewhat	14 (17)	13 (16)	27 (17)	
Much	31 (37)	21 (26)	52 (32)	
Very much	12 (14)	15 (18)	27 (16)	0.177
Total	84	82	166	

*P-*value is based on a Chi-square statistical comparison

### Temporal variation in perceived mosquito nuisance

**Fig. 2:**
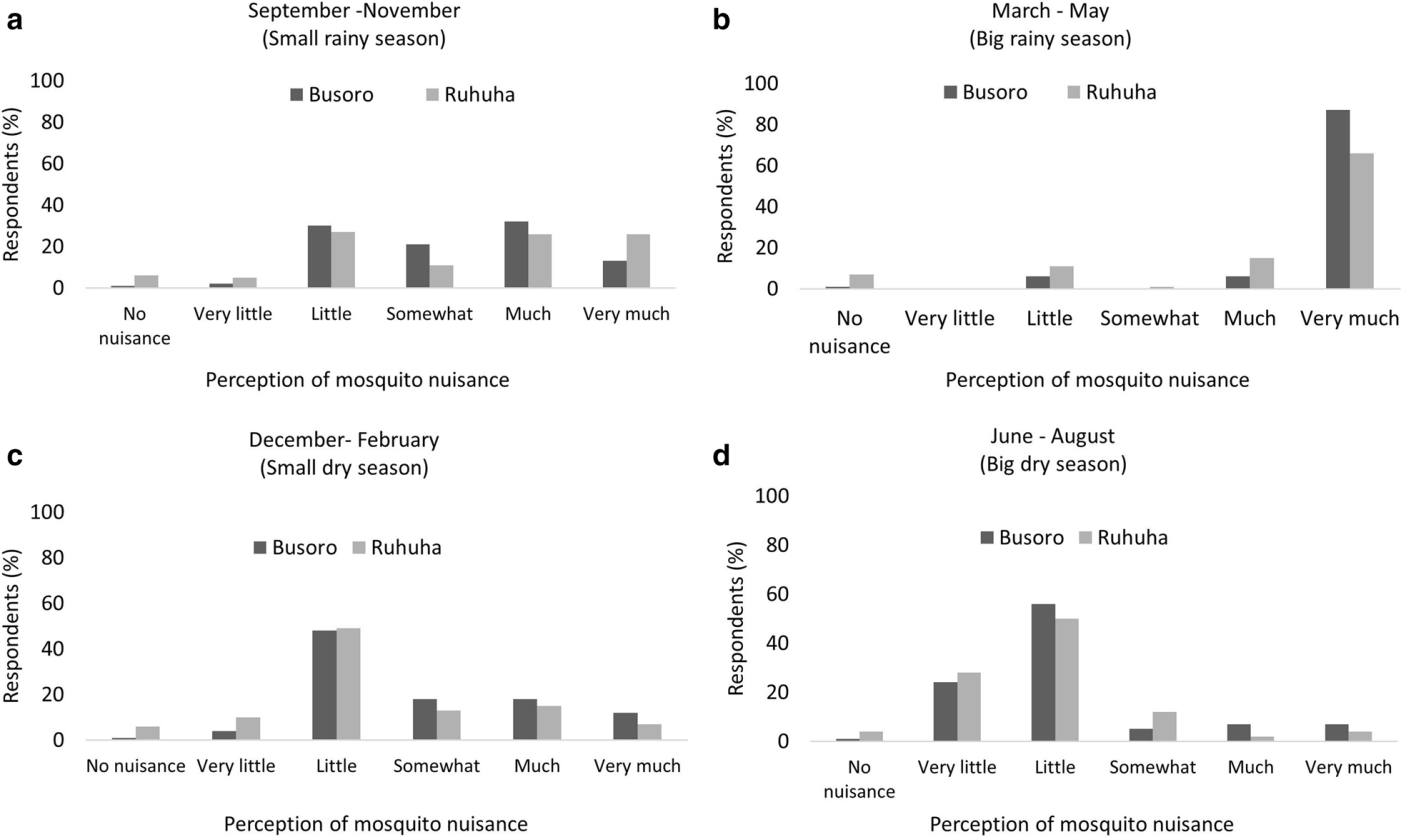
Mosquito nuisance as experienced during the four seasons reported by 166 respondents from Busoro and Ruhuha sector, Rwanda

When asked in what season respondents perceive mosquito nuisance, most respondents reported to experience “very much” nuisance during the long rainy season (March–May; Fig. 2b). Interestingly, more respondents in Busoro (n = 73) perceived mosquito nuisance in this season than in Ruhuha (n = 54). Respondents perceived “little” to “very much” nuisance during the short rainy season (September until November; Fig. 2a). Respondents mostly perceived “some” to “little” nuisance during the small rainy season (December-February; Fig. 2c) and mostly “very little” to “little” in the big dry season (June until August; Fig. 2d).

### Spatial variation in perceived mosquito nuisance

**Fig. 3:**
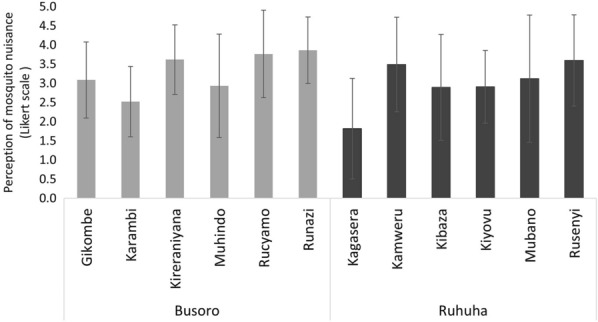
Perceived mosquito nuisance in 12 villages in Busoro and Ruhuha sector, Rwanda

Within each sector, there was substantial spatial variation at village level in the degree of mosquito nuisance perceived indoors (Fig. 3). In Busoro, the highest perceived mosquito nuisance was from Runazi (x̅ = 3.9), Rucyamo (x̅ = 3.8), and Kireranyana (x̅ = 3.6), followed by Gikombe (x̅ = 3.1), Muhindo (x̅ = 2.9) and Karambi (x̅ = 2.5) (Fig. 3). In Ruhuha, 82 households from Kamweru (x̅ = 3.5) and Rusenyi (x̅ = 3.6) reported to experience much nuisance in their houses while the remaining participants from Kibaza (x̅ = 2.9), Kiyovu (x̅ = 2.9), and Mubano (x̅ = 3.1) reported having experienced some nuisance. Interestingly, respondents from Kagasera reported having experienced little mosquito nuisance (x̅ = 1.8) during the period of study.

### Relationship between mosquito abundance and perceived indoor mosquito nuisance

First, a Spearman correlation coefficient was computed to assess the relationship between the number of mosquitoes (total Culicidae and total *An. gambiae s.l*.) collected and the perceived nuisance level experienced in the houses by the participants. When data from both years and sites were aggregated, there was a moderate but significant correlation between the total Culicidae and perceived nuisance indoors (r_s_ = 0.316, n = 166, *P* < 0.001). Similarly, there was a moderate, but significant correlation between the number of *An. gambiae s.l*. collected indoors and the perceived mosquito nuisance (r_s_ = 0.281, n = 166, *P* < 0.001).

**Fig. 4:**
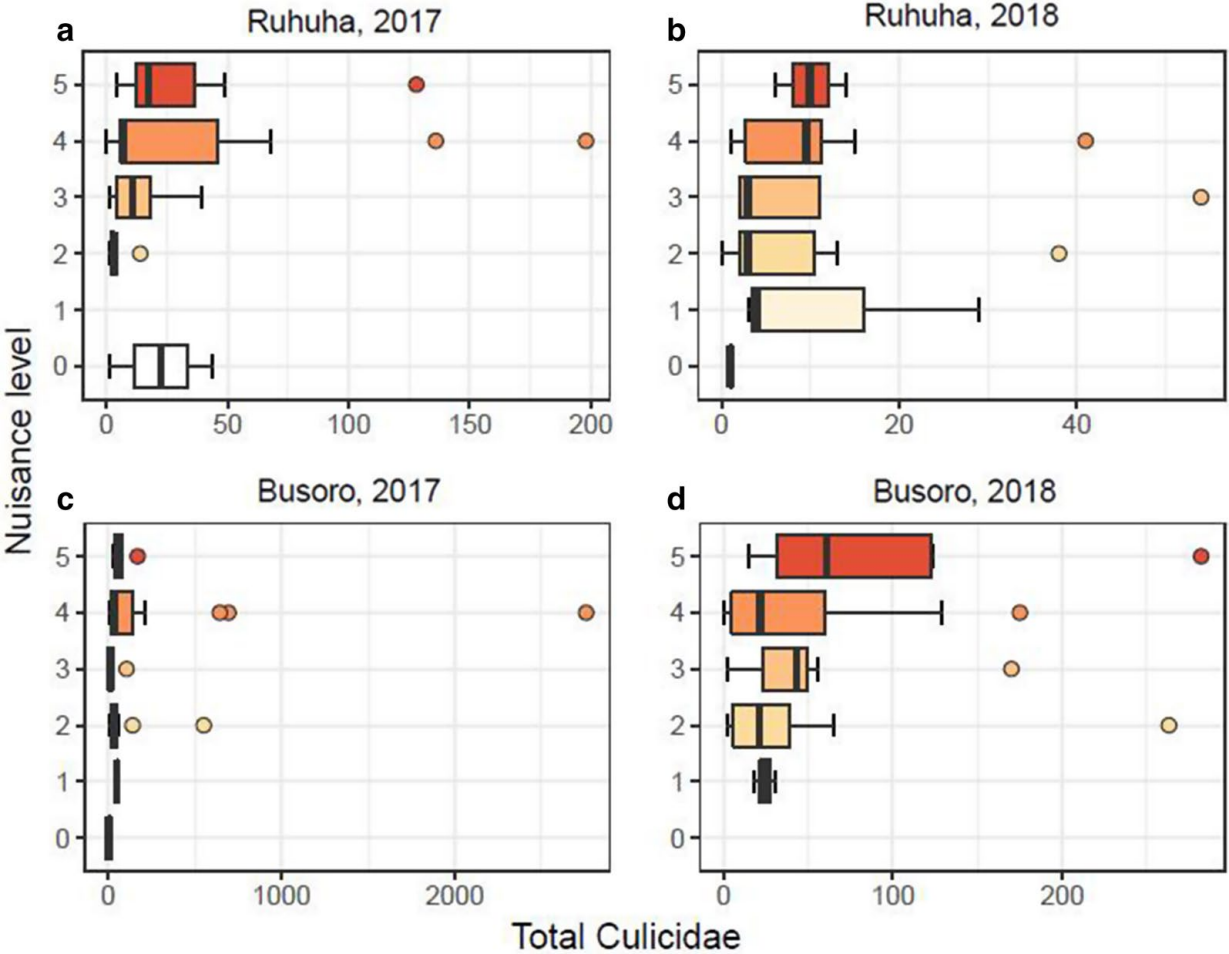
Boxplots showing the distribution of total mosquito (Culicidae) densities for each nuisance level reported per household in the two years (2017 and 2018) and two sectors (Ruhuha and Busoro) of study. Note the different scales of the x-axes for each panel

When the data were analysed per year and per sector for Culicidae, there was a strong spatial difference. No significant correlation was observed between total mosquito numbers and perceived nuisance indoors in Busoro for both years (2017: r_s_ = 0.055, n = 42, *P* = 0.731 and 2018: r_s_ = 0.200, n = 42, *P* = 0.204; Fig. 4c, d), whereas there were moderate, but significant correlations between the total mosquito numbers and perceived mosquito nuisance in Ruhuha for both years of study (2017: r_s_ = 0.389, n = 40, *P* = 0.013 and 2018: r_s_ = 0.305, n = 42, *P* = 0.049; Fig. 4a, b; Table 8).

**Table 8:** Spearman correlation coefficients between perceived level of mosquito nuisance and number of mosquitoes (*Anopheles gambiae* only, or total Culicidae)

Site	Year	Mosquito group	r_s_	n	*P*
Busoro	2017	Culicidae	0.055	42	0.731
Busoro	2018	Culicidae	0.200	42	0.204
Ruhuha	2017	Culicidae	0.389	40	*0.013*
Ruhuha	2018	Culicidae	0.305	42	*0.049*
Busoro	2017	*An. gambiae s.l.*	0.370	42	*0.016*
Busoro	2018	*An. gambiae s.l.*	0.119	42	0.452
Ruhuha	2017	*An. gambiae s.l.*	0.450	40	*0.004*
Ruhuha	2018	*An. gambiae s.l.*	0.251	40	0.109

Significant *P*-values are indicated in italics

**Fig. 5:**
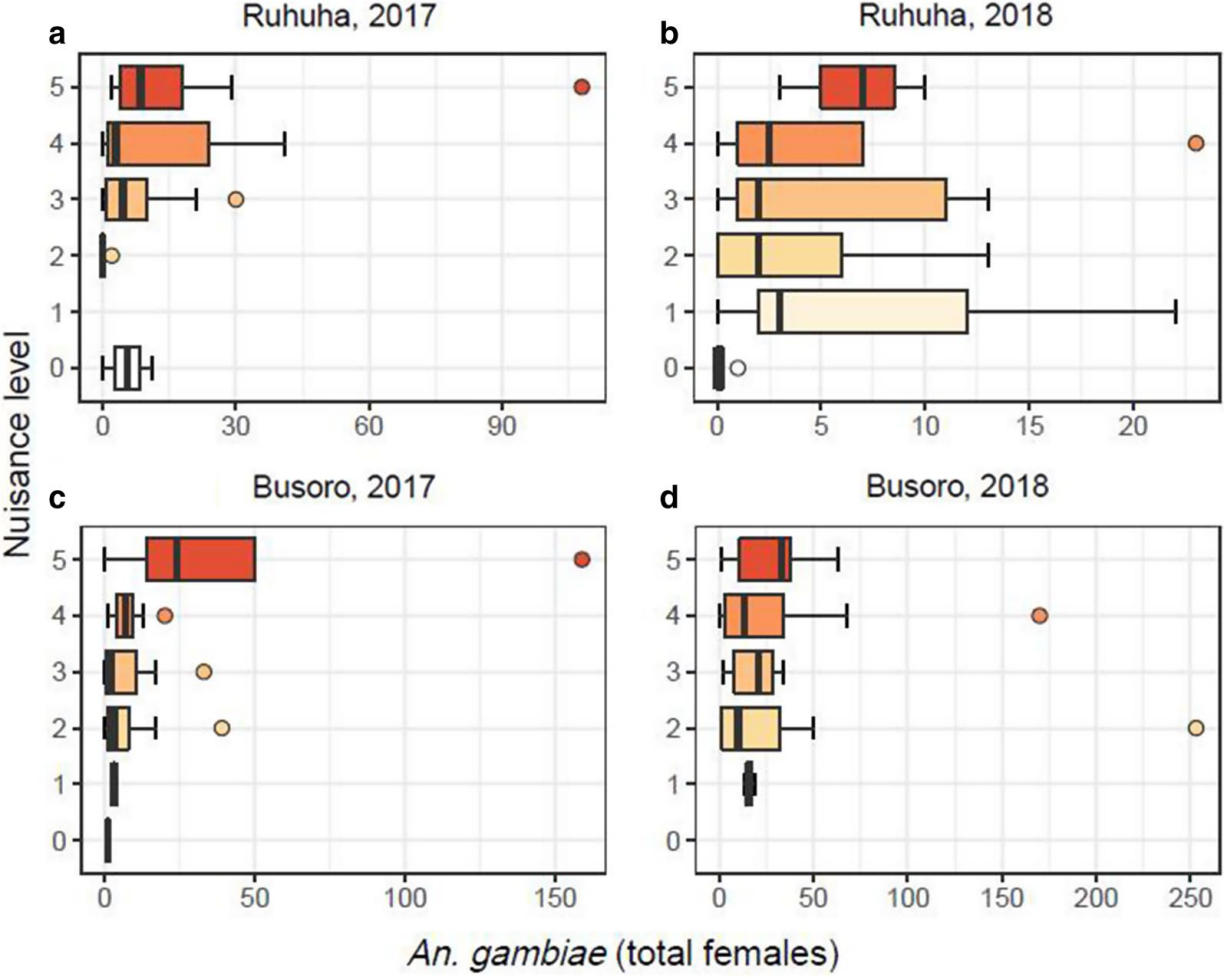
Boxplots showing the distribution of *Anopheles gambiae* densities for each nuisance level reported per household in the two years (2017 and 2018) and two sectors (Ruhuha and Busoro) of study. Note the different scales of the x-axes for each panel

For *An. gambiae s.l.* per sector and year, there was a strong temporal difference. There were significant correlations in both sectors in 2017 (Busoro: r_s_ = 0.37, n = 42, *P* = 0.016; Ruhuha: r_s_ = 0.45, n = 40, *P* = 0.004; Fig. 5a, c). However, in 2018, these significant correlations between perceived nuisance and *An. gambiae s.l.* were absent for both sectors (Busoro: r_s_ = 0.119, n = 42, *P* = 0.452; Ruhuha: r_s_ = 0.251, n = 40, *P* = 0.109; Fig. 5b, d; Table 8).

**Fig. 6:**
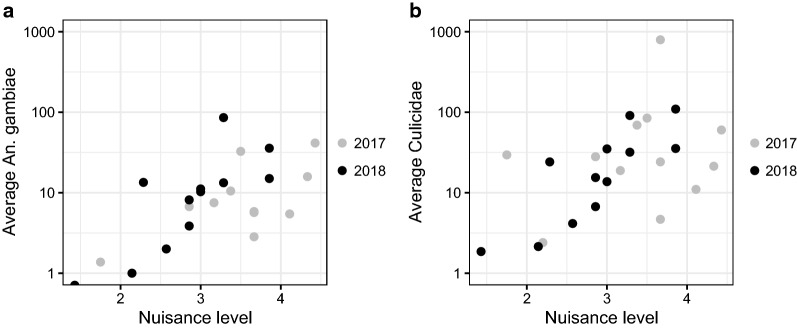
Scatterplots showing the correlation between average level of mosquito nuisance reported and average number of mosquitoes at village level (*An. gambiae s.l.*, **a**; Total Culicidae, **b**). Each dot represents the average for one village consisting of 6–8 sampled households (2017: grey dots, 2018: black dots). Please note the logarithmic scale of the y-axis in both panels

When data were analysed one spatial level lower, i.e., by village, there were significant correlations between average nuisance level reported and *An. gambiae s.l.* (r_s_ = 0.607, *P* = 0.002, Fig. 6a), as well as between average nuisance level and total mosquitoes (r_s_ = 0.528, *P* = 0.008, Fig. 6b). When analysed per year separately, the correlations were strong and significant for 2018 (black dots, Fig. 6), but not significant for the data from 2017 (grey dots, Fig. 6).

## Discussion

The mosquito nuisance derived from the questionnaires revealed a significant nuisance caused by mosquitoes, notably from the Culicidae. The findings show that there was a high mosquito density including *Culex* species and mainly *An. gambiae s.l.* for both years in both sectors. A higher density of mosquitoes and malaria vectors were partially explained by the house construction materials for roof and walls, as well as by the presence of eaves and number of occupants in the house. These factors also contribute to a high level of perceived mosquito nuisance, although correlations were space (sector) and time (year) dependent. The presence of *P. falciparum-*infected mosquitoes in the houses, as shown for the 2017 data, contributes to the risk of contracting malaria. The results are supported by several other studies that show that individuals who live in rural areas in poorly constructed houses are exposed to more mosquito bites, and hence to an increased risk of malaria transmission [26–31]. In a study conducted in Kenya, houses made of wood and mud exhibited a significant effect on mosquito abundance in the houses [32]. Factors such as closed eaves reduced rates of house entry by anopheline mosquitoes compared to fully open eaves as was also demonstrated in other studies from Tanzania, The Gambia and Kenya [33–37].

Spatio-temporal variations in mosquito abundance were observed between Busoro and Ruhuha. In Busoro, mosquitoes, including numbers of *An. gambiae s.l*., were collected in significantly higher numbers in comparison with Ruhuha. This spatial difference may be explained by the fact that although both sectors have wetlands, Busoro is characterized by a larger area (178 ha) than Ruhuha (93 ha) that is dedicated to rice irrigation [14]. The difference was strongly influenced by collections from one of the villages (Rucyamo), which is the village closest to the irrigated fields (Fig. 1) and which contributed to 65% of all mosquitoes collected. This village was also the village with the next highest level of mosquito nuisance reported. Living near the rice field, the chance of more mosquitoes and to experience more nuisance was higher because the wetlands provide good mosquito breeding habitat [38, 39].

In the present study, the predominant sibling species of the *An. gambiae* complex was *An. gambiae s.s.* for both years and sectors combined (66%). This dominance of *An. gambiae s.s.* is similar to a study conducted in one site near Kigali City in 2007 by President’s Malaria Initiative-Rwanda, in which it was reported that *An. gambiae s.s*. accounted for 93.6% of the total of 157 *An. gambiae s.l.* examined by PCR, while *An. arabiensis* accounted for the remaining 6.4% [40]. However, the finding was contrary to a study where the characterization of *An. gambiae s.l.* from 10 sentinel sites revealed that the predominant sibling species was *An. arabiensis* (83%) [41]. Although *An. funestus* was recognized as the dominant *Anopheles* species in previous studies from Rwanda [42, 43], this species was collected only in Rucyamo, the village closest to the more permanently inundated wetlands and rice fields, which are ideal habitats for this species.

For 2017, the *P. falciparum* infection rate in Busoro was higher compared to the infection rate in Ruhuha. Other anopheline species, though collected in low numbers, should not be neglected in strategies for malaria control and elimination, because they can transmit other mosquito-borne diseases, such as the Babanki virus (BBKV) that is transmitted by *An. brohieri,* as found in Senegal [44]. Albeit in low numbers, both *An. pharoensis* and *An. ziemanni* can transmit *P. falciparum* as observed in studies from Cameroon, Ethiopia, Guinea Bissau, Tchad, and Kenya, respectively [38, 39, 45–47]. Although *Culex* spp. have not been incriminated as vector of disease in Rwanda, the species caused a high burden of nuisance in Cameroon [48]. Considering this group of species as potential vector for other vector-borne diseases will be important in the framework of integrated vector management. Reducing their numbers would substantially reduce mosquito nuisance experienced, and thus enhance community involvement in uptake of vector control for malaria prevention [49].

The respondents experienced mosquitoes as a significant problem in their daily life, especially during the rainy season that lasts from March to May. The increase in perception of mosquito nuisance over the seasons corresponds to the increase of vector density at the start of the long rainy season, which is mainly due to an increase in wetland area providing suitable habitats for larval development of mosquitoes. The percentage of participants that reported much nuisance was higher in Busoro (87%) than in Ruhuha (67%). Living in the vicinity of marshlands increased the chance to experience higher mosquito abundance and hence higher nuisance level as observed from participants from Busoro and Ruhuha [13, 14]. In addition, the presence of blocked ditches produced by exploitation of sand for house construction was noted. Such ditches are known to be artificial mosquito breeding sites [50, 51].

The correlations found between nuisance and number of mosquitoes can be explained as larger numbers of mosquitoes collected indoors will result in more biting activity and, hence a higher level of mosquito nuisance. The results suggest that levels of perceived mosquito nuisance are in some way indicative of mosquito densities indoors. Consequently, it could be argued that perceived mosquito nuisance in the peridomestic area can be used as a global indicator for malaria transmission risk. Such data can be obtained by filling out a questionnaire indicating the level of nuisance expressed on a Likert scale. A study conducted in Algeria demonstrated that perception of citizens can help to identify occurrence of *Aedes albopictus* in a residential neighbourhood in Bir-Khadem [52]. This helped to put in place vector control measures that could prevent the propagation of *Ae. albopictus* to other areas and to avoid the massive use of insecticides for vector control, which could ultimately lead to insecticide resistance [52].

It should be noted that when considering each sector separately, perceived mosquito nuisance was significantly correlated to the numbers of *An. gambiae* when data from both years were added together, while for Culicidae, perceived mosquito nuisance was correlated to the number of Culicidae for Ruhuha but not for Busoro, even after adding datasets of both study years together. The reasons why there was no correlation for Busoro remains unclear. This may be explained by the fact that in Busoro mosquito densities were more extreme than in Ruhuha and that variation in nuisance by these high densities could no longer be caught in the level of perceived mosquito nuisance.

We have shown that data on mosquito nuisance can indicate malaria vector abundance and hence identify malaria transmission risk. An important next step, however, is to scale-up the approach, validate it in other settings and possibly integrate it in vector surveillance efforts. ICT-based technologies, such as apps and web-based platforms, can greatly support such initiatives, as evidenced by the success of the Mosquito Alert application in Spain [6]. This requires investment costs upfront (ICT, human resources etc.), but these may largely be paid off by boosting vector surveillance data collection, as well as by building durable partnerships between public health authorities and citizens [53].

## Conclusions

Poor housing construction significantly led to increased malaria vector density and thus possibly malaria risk in rural Rwanda. This suggests that good house construction needs to be considered as one of the vector control strategies that can be provided for poor populations. At the largest scale in this study, i.e., if data for years and sectors are combined, the relationships between the level of perceived mosquito nuisance and mosquito density at family and species level were clearly shown. Perception of mosquito nuisance denoted in a questionnaire survey could be used as an indicator of mosquito abundance and, hence, for *An. gambiae s.l.* occurrence. Involving citizens in reporting the level of mosquito nuisance can contribute to improved malaria vector surveillance and control.

## Supplementary Information

**Additional file 1.** Mosquito species collected using CDC light traps in selected villages in Busoro and Ruhuha sector, Rwanda (2017)

**Additional file 2.** Mosquito species collected using CDC light traps in selected villages in Busoro and Ruhuha sector, Rwanda (2018)

**Additional file 3.** Members of the Anopheles gambiae complex found among samples of Anopheles gambiae s.l. tested from Ruhuha and Busoro sector, Ruhuha

## Data Availability

Data supporting the conclusions of this article are included within the article. The datasets used and/or analysed during the present study are available from the corresponding author upon reasonable request.

## Abbreviations

CDC: Centers for Disease Control
EIR: Entomological inoculation rate
HH: Household
ODK: Open Data Kit
PBS: Phosphate Buffered Saline solution
ELISA: Enzyme-Linked ImmunoSorbent Assay
IgG: Immunoglobulin G
CSP: Circumsporozoite protein
OD: Optical density
PCR: Polymerase chain reaction
GLM: Generalized linear model
PMI: President’s malaria initiative
