# Supplementary material for: Monitoring mosquito nuisance for the development of a citizen science approach for malaria vector surveillance in Rwanda
Source: Malar J. 2021 Jan 10;20:36. doi: 10.1186/s12936-020-03579-w (PMC7798336; doi:10.1186/s12936-020-03579-w)
Supplement: Supplementary file 1 — Additional file 1. Mosquito species collected using CDC light traps in selected villages in Busoro and Ruhuha sector, Rwanda (2017) [file 12936_2020_3579_MOESM1_ESM.docx]

**Additional file 1 -** Mosquito species collected using CDC light traps in selected villages in Busoro and Ruhuha sector, Rwanda (2017).

| **Mosquito species collected** | **Busoro** | | | | | | **Ruhuha** | | | | | | **TOTAL** | **Species composition %** |
| --- | --- | --- | --- | --- | --- | --- | --- | --- | --- | --- | --- | --- | --- | --- |
|  | Gikombe | Karambi | Kireranyana | Muhindo | Rucyamo | Runazi | Kagasera | Kamweru | Kibaza | Kiyovu | Mubano | Rusenyi |  |  |
| *An. gambiae* s.l. | 45 | 11 | 84 | 47 | 34 | 290 | 3 | 49 | 261 | 17 | 35 | 95 | 971 | 13.2 |
| *An. maculipalpis* | 0 | 0 | 0 | 0 | 1 | 0 | 0 | 3 | 4 | 0 | 0 | 5 | 13 | 0.2 |
| *An. pharoensis* | 0 | 0 | 0 | 0 | 6 | 0 | 0 | 0 | 1 | 0 | 0 | 0 | 7 | 0.1 |
| *An. rufipes* | 0 | 11 | 0 | 0 | 1 | 1 | 0 | 0 | 0 | 0 | 0 | 0 | 13 | 0.2 |
| *An. ziemanni* | 0 | 0 | 0 | 0 | 42 | 0 | 0 | 0 | 0 | 0 | 0 | 0 | 42 | 0.6 |
| Total *Anopheles* spp | 45 | 22 | 84 | 47 | 84 | 291 | 3 | 52 | 266 | 17 | 35 | 100 | 1046 | 14.2 |
| *Culex* spp | 68 | 213 | 469 | 149 | 4678 | 129 | 9 | 40 | 410 | 11 | 110 | 28 | 6314 | 85.7 |
| *Mansonia* spp | 0 | 1 | 0 | 0 | 1 | 1 | 0 | 7 | 0 | 0 | 0 | 0 | 10 | 0.1 |
| Total Culicinae spp | 68 | 214 | 469 | 149 | 4679 | 130 | 9 | 47 | 410 | 11 | 110 | 28 | 6324 | 85.8 |
| Total Culicidae spp | 113 | 236 | 553 | 196 | 4763 | 421 | 12 | 99 | 676 | 28 | 145 | 128 | 7370 | 100.0 |
| % *Anopheles* spp | 39.8 | 9.3 | 15.2 | 24.0 | 1.8 | 69.1 | 25.0 | 52.5 | 39.3 | 60.7 | 24.1 | 78.1 | 14.2 |  |
