# Supplementary material for: Monitoring mosquito nuisance for the development of a citizen science approach for malaria vector surveillance in Rwanda
Source: Malar J. 2021 Jan 10;20:36. doi: 10.1186/s12936-020-03579-w (PMC7798336; doi:10.1186/s12936-020-03579-w)
Supplement: Supplementary file 2 — Additional file 2. Mosquito species collected using CDC light traps in selected villages in Busoro and Ruhuha sector, Rwanda (2018) [file 12936_2020_3579_MOESM2_ESM.docx]

**Additional file 2 -** Mosquito species collected using CDC light traps in selected villages in Busoro and Ruhuha sector, Rwanda (2018).

| **Mosquito species collected** | **Busoro** | | | | | | **Ruhuha** | | | | | | **TOTAL** | **Species composition %** |
| --- | --- | --- | --- | --- | --- | --- | --- | --- | --- | --- | --- | --- | --- | --- |
|  | Gikombe | Karambi | Kireranyana | Muhindo | Rucyamo | Runazi | Kagasera | Kamweru | Kibaza | Kiyovu | Mubano | Rusenyi |  |  |
| *An. brohieri* | 0 | 0 | 0 | 0 | 1 | 1 | 0 | 0 | 0 | 1 | 0 | 1 | 4 | 0.2 |
| *An. funestus* | 0 | 0 | 0 | 0 | 31 | 0 | 0 | 0 | 0 | 0 | 0 | 0 | 31 | 1.2 |
| *An. gambiae* s.l. | 72 | 93 | 105 | 78 | 250 | 600 | 0 | 27 | 94 | 7 | 14 | 57 | 1397 | 53.8 |
| *An. maculipalpis* | 0 | 0 | 0 | 1 | 2 | 1 | 0 | 0 | 5 | 0 | 1 | 4 | 14 | 0.5 |
| *An. pharoensis* | 0 | 0 | 0 | 1 | 7 | 0 | 0 | 0 | 0 | 0 | 0 | 0 | 8 | 0.3 |
| *An. ziemanni* | 0 | 0 | 0 | 0 | 14 | 0 | 0 | 0 | 0 | 0 | 0 | 0 | 14 | 0.5 |
| Total *Anopheles* spp | 72 | 93 | 105 | 80 | 305 | 602 | 0 | 27 | 99 | 8 | 15 | 62 | 1468 | 56.6 |
| *Culex* spp | 24 | 127 | 141 | 165 | 449 | 35 | 13 | 20 | 70 | 7 | 14 | 46 | 1111 | 42.8 |
| *Mansonia* spp | 0 | 3 | 2 | 0 | 11 | 0 | 0 | 0 | 0 | 0 | 0 | 0 | 16 | 0.6 |
| Total Culicinae spp | 24 | 130 | 143 | 165 | 460 | 35 | 13 | 20 | 70 | 7 | 14 | 46 | 1127 | 43.4 |
| Total Culicidae | 96 | 223 | 248 | 245 | 765 | 637 | 13 | 47 | 169 | 15 | 29 | 108 | 2595 | 100.0 |
| % *Anopheles* spp | 75.0 | 41.7 | 42.3 | 32.7 | 39.9 | 94.5 | 0.0 | 57.4 | 58.6 | 53.3 | 51.7 | 57.4 | 56.6 |  |
