# Supplementary material for: Monitoring mosquito nuisance for the development of a citizen science approach for malaria vector surveillance in Rwanda
Source: Malar J. 2021 Jan 10;20:36. doi: 10.1186/s12936-020-03579-w (PMC7798336; doi:10.1186/s12936-020-03579-w)
Supplement: Supplementary file 3 — Additional file 3. Members of the Anopheles gambiae complex found among samples of Anopheles gambiae s.l. tested from Ruhuha and Busoro sector, Ruhuha [file 12936_2020_3579_MOESM3_ESM.docx]

**Additional file 3 -** Members of the *An. gambiae* complex found among samples of *An. gambiae* s.l. tested from Ruhuha and Busoro sector, Ruhuha

| **Sibling species** | **2017** | | | | | | **2018** | | | | | | **TOTAL** | |
| --- | --- | --- | --- | --- | --- | --- | --- | --- | --- | --- | --- | --- | --- | --- |
|  | Busoro | | Ruhuha | | *S/total* | | Busoro | | Ruhuha | | *S/total* | |  |  |
|  | n | % | n | % | n | % | n | % | n | % | n | % | n | % |
| *An. arabiensis* | 31 | 36.0 | 3 | 3.5 | 34 | 39.5 | 25 | 16.7 | 15 | 10.0 | 40 | 26.7 | 74 | 31.4 |
| *An. gambiae* s.s. | 13 | 15.1 | 31 | 36.0 | 44 | 51.2 | 96 | 64.0 | 5 | 3.3 | 101 | 67.3 | 145 | 61.4 |
| Not amplified | 4 | 4.7 | 3 | 3.5 | 7 | 8.1 | 6 | 4.0 | 3 | 2.0 | 9 | 6.0 | 16 | 6.8 |
| Contaminated | 1 | 1.2 | 0 | 0.0 | 1 | 1.2 | 0 | 0.0 | 0 | 0.0 | 0 | 0.0 | 1 | 0.4 |
| TOTAL | 49 | 57.0 | 37 | 43.0 | 86 | 100.0 | 127 | 84.7 | 23 | 15.3 | 150 | 100.0 | 236 | 100.0 |
